# Stroke in the state of Alagoas, Brazil: a descriptive analysis of a northeastern scenario

**DOI:** 10.1590/0004-282X-ANP-2021-0194

**Published:** 2022-08-08

**Authors:** Letícia Januzi de Almeida Rocha, Kauan Araújo da Silva, Arthur de Lima Chagas, Arthur de Oliveira Veras, Vitor Gustavo Leão Souto, Maria Clara Motta Barbosa Valente, Jussara Almeida de Oliveira Baggio

**Affiliations:** 1 Universidade Federal de Alagoas, Faculdade de Medicina, Maceió AL, Brazil.; 2 Universidade Estadual de Ciências da Saúde de Alagoas, Maceió AL, Brazil.; 3 Universidade Federal de Alagoas, Curso de Medicina, Arapiraca AL, Brazil.

**Keywords:** Stroke, Epidemiology, Risk Factors, Functional Status, Acidente Vascular Cerebral, Epidemiologia, Fatores de Risco, Estado Funcional

## Abstract

**Background:**

There is little information available on stroke epidemiology in the northeast of Brazil.

**Objective:**

Our objective was to investigate the prevalence of the stroke subtypes, prevalence of cerebrovascular risk factors and patterns of management in a public neurovascular outpatient referral service, in Alagoas.

**Methods:**

Data were prospectively collected from consecutive patients with stroke who were treated in a specialized neurovascular clinic between November 2016 and June 2018. Recurrence was evaluated by telephone 12 months after patients had been included in the study.

**Results:**

We evaluated 190 patients (mean age, 60.22 ( 13.29 years; 60.5% males). Ischemic stroke was the most frequent subtype (85.2%). Sedentary lifestyle was the most common risk factor (71.6%), followed by hypertension (62.6%) and stroke family history (41.1%). Only 21.5% of the patients were transported by ambulance to the hospital, and 42.6% received medical support in hospital units or emergency units with no imaging support. The median NIHSS was 2.5 (IQR, 1-5) and mRS was 2 (IQR, 1-3). We found a high rate of undetermined stroke (35.8%), and few patients completed the etiological investigation. One year after inclusion in the study, 12 patients (6.3%) had died and 14 (7.3%) had had another stroke.

**Conclusions:**

The prevalence of cerebrovascular risk factors and clinical presentation were similar to what had been seen in previous series. A notable number of patients received medical support in institutions with no imaging equipment. The high number of cases of undetermined stroke etiology shows the difficulty in accessing healthcare services in Alagoas.

## RESUMO

**Antecedentes:**

Até o momento existe pouca informação disponível na literatura sobre a epidemiologia do acidente vascular verebral (AVC) no nordeste brasileiro.

**Objetivo:**

Investigar a prevalência dos subtipos de AVC, dos fatores de risco para doenças cerebrovasculares e o manejo do AVC em um serviço público especializado em Alagoas.

**Método:**

Os dados foram coletados de forma prospectiva e consecutiva de pacientes com diagnóstico de AVC em um ambulatório especializado em neurovascular, de novembro de 2016 a junho de 2018. Recorrência do AVC foi avaliada por telefone 12 meses após a inclusão no estudo.

**Resultados:**

Foram avaliados 190 pacientes, idade média de 60,22(13,29 anos, 60,5% homens. AVC isquêmico foi o subtipo mais comum (85,2%). Sedentarismo foi o fator de risco mais prevalente (71,6%), seguido de hipertensão (62,6%) e história familial de AVC (41,1%). Somente 21,5% dos pacientes foram transportados por ambulância até o hospital e 42,6% receberam o primeiro atendimento em serviço médico sem suporte de exame de imagem. A mediana do NIHSS foi 2,5 (IQR, 1-3). Encontramos alta prevalência de AVC indeterminado (35,8%) e poucos pacientes completaram a investigação etiológica. Após um ano da inclusão no estudo, 12 pacientes (6,3%) morreram e 14 (7,3%) tiveram outro AVC.

**Conclusão:**

A prevalência dos fatores de risco para doenças cerebrovasculares e a apresentação clínica foram similares a séries prévias. Um número expressivo de pacientes recebeu atendimento médico em locais sem exames de imagem. Houve alto número de pacientes com AVC indeterminado, o que mostra a dificuldade de acesso ao sistema de saúde em Alagoas.

## INTRODUCTION

Cerebrovascular diseases, including stroke, are one of the leading causes of mortality in most Latin American countries^1^. In Brazil, the incidence of stroke is 139.91/100,000 inhabitants. Stroke has a huge social impact, with an estimated loss of 1437.74 years of healthy life, and its treatment is costly for the Brazilian healthcare system^2^ ^-^ ^4^.

Several studies have been developed to characterize the prevalence, incidence and clinical characteristics of stroke patients in Brazil^5^ ^-^ ^8^. However, regional differences are significant. In the northeastern region, the stroke age-standardized mortality rate is high. In comparison with other state capitals in the northeastern region, Maceió had the highest mortality rate and the lowest human development index (HDI)^9^. A cohort study conducted in Fortaleza, also located in the northeastern region, demonstrated that investigation of stroke etiology was less common and that the frequency of post-stroke disability was higher than in other national and international studies^5^. Hence, regional studies are helpful for understanding the clinical characteristics of stroke patients and for improving the management of this disease.

The objective of this study was to investigate the prevalence of the stroke subtypes, the prevalence of cerebrovascular risk factors and the patterns of management in a public neurovascular outpatient referral service, in Alagoas.

## METHODS

### Study population

We evaluated patients with cerebrovascular disease, confirmed by means of neuroimaging, who were consecutively treated in a public specialized neurovascular clinic in Alagoas, Brazil, between November 2016 and June 2018. All the patients were over 18 years old. Patients were excluded if they had another neurological condition or any severe concomitant systemic illness. 

The ethics committee of the Federal University of Alagoas approved this study and written informed consent was obtained from all of the participants. 

### Study protocol

The protocol consisted of assessment of demographic and clinical data, including use of the Bamford classification, which classifies stroke as lacunar syndrome (LACS), partial anterior circulation syndrome (PACS), total anterior circulation syndrome (TACS) and posterior circular syndrome (POCS)^10^; and use of the National Institutes of Health Stroke Scale (NIHSS)^11^ and the modified Rankin Scale (mRS)^12^. 

Risk factors, including hypertension, diabetes mellitus, smoking, alcoholism, dyslipidemia, atrial fibrillation, coronary artery disease, prior stroke or TIA, were registered upon inclusion of patients in the study. Hypertension, diabetes mellitus and dyslipidemia were defined as histories of prior or current use of appropriate medications for these conditions. Smoking and alcohol habits were defined as current use in the year of the stroke or one year before the ictus. Coronary artery disease was defined as a history of angina, acute myocardial infarction or coronary revascularization. Atrial fibrillation was defined from known previous diagnoses or through new evidence from Holter monitoring or electrocardiogram. 

We also registered any complementary examinations that the patients had undergone, in order to enable TOAST (*Trial of Org 10172 in Acute Stroke Treatment)* classification^13^. Recurrence was evaluated by means of telephone calls 12 months after patients had been included in the study. 

### Statistical analysis

The analyses were performed using the SPSS software (version 20.0; Chicago, IL, United States) at a significance level of 5%. Continuous variables were summarized as means and standard deviations or as medians and interquartile ranges (IQR). Categorical variables were presented as percentages. We also compared groups of ischemic versus hemorrhagic stroke and of female versus male patients, using the Mann-Whitney test and χ^2^ test.

## RESULTS

We included 190 patients with a mean age of 60.22 ( 13.29 years, and 115 (60.5%) were males. The mean length of time from stroke to inclusion in the study was 27.2 ± 33.1 months. Table 1 describes the demographic characteristics and risk factors of all the patients and according to gender.

**Table 1.** Demographic characteristics and risk factors of all patients according to sex.

Characteristics		Total n = 190 (100%)	Males n = 115 (60.5%)	Females n = 75 (39.5%)	p-value
Age, mean (± SD)		60.22 (13.29)	60.33 (1.10)	60.02 (1.76)	0.93
Ethnicity (%)^1^	White	29 (15.3)	21 (18.2)	8 (10.6)	0.34
	Brown	115 (60.5)	68 (59.1)	47 (62.6)	
	Black	39 (20.5)	23(20)	16 (21.3)	
	Indigenous	2 (1.1)	1(0.8)	1 (1.3)	
	Not declared	5 (2.63)	2 (1.7)	2 (2.6)	
Marital status (%)	Married	118 (62.1)	89 (77.3)	29 (38.6)	0.0001^#^
	Single	30 (15.8)	10 (8.6)	20 (26.6)	
	Widower	27 (14.2)	8 (6.9)	19 (25.3)	
	Divorced	15 (7.9)	8 (6.9)	7 (9.3)	
Years of schooling (%)	Illiterate	38 (20)	19 (16.5)	18 (24)	0.57
	1-4 years	48 (25.3)	30 (26.08)	19 (25.3)	
	5-9 years	59 (31.1)	36 (31.3)	23 (30.6)	
	10-12 years	27 (14.2)	16 (13.9)	11 (14.6)	
	13 years or more	16 (8.4)	12 (10.4)	4 (5.3)	
	Not declared	2 (1.1)	2 (1.7)	0	
Occupation (%)^2^	Employed	18 (9.5)	14 (12.1)	4 (5.3)	0.001^#^
	Unemployed	25 (13.2)	15 (13.04)	10 (13.3)	
	Government beneficiary	36 (18.9)	25 (21.7)	11 (14.6)	
	Pensioner	4 (2.1)	0	4 (5.3)	
	Retired	95 (50)	60 (52.1)	35 (46.6)	
	Student	3 (1.6)	1 (0.8)	2 (2.6)	
	Housewife	9 (4.7)	0	9 (12)	
Income (%)^3^ (in minimum monthly wages)	Up to 1	73 (38.4)	38 (33)	35 (46.6)	0.001^#^
	2 to 3	93 (48.9)	57 (49.5)	36 (48)	
	> 3	17 (8.9)	16 (13.9)	1 (1.3)	
	Not declared	7 (3.6)	4 (3.4)	3 (4)	
Risk factors (%)	Sedentary lifestyle (yes)	136 (71.6)	81 (70.4)	55 (73.3)	0.63
	Hypertension (yes)	119 (62.6)	71 (61.7)	48 (64)	0.93
	Stroke familiar history (yes)	78 (41.1)	48 (41.7)	30 (40)	0.77
	Prior stroke or TIA (yes)	59 (31.1)	32 (27.8)	21 (28)	0.98
	Diabetes (yes)	56 (29.5)	38 (33)	18 (24)	0.40
	Smoker (yes)	40 (21.1)	25 (21.7)	15 (20)	0.72
	Alcoholism (yes)	37 (19.5)	33 (28.6)	4 (5.3)	0.0001^#^
	Cardiomyopathy (yes)	23 (12.1)	12 (10.4)	11 (14.6)	0.29
	Atrial fibrillation (yes)	16 (8.4)	5 (4.3)	11 (14.6)	0.009^#^
	Chagas disease (yes)	12 (6.3)	7 (6.08)	5 (6.6)	0.77

SD: standard deviation; TIA: transient ischemic attack; ^1^: for the (^2^ test, the participants were classified as white, brown or others; ^2^: for the (^2^ test, the participants were classified as employed, unemployed, beneficiaries or without income (students and housewives); ^3^: for the (^2^ test, the participants were classified as up to 1 minimum monthly wage, 2 to 3 wages or > 3 wages; ^#^(^2^ test: significance level < 0.05.

The majority of the patients (78.8%) had access to medical assistance on the first day of the event. However, only 21.5% were transported by ambulance to the hospital, 42.6% received medical support in hospital units or emergency units with no imaging support and only 2.6% received acute reperfusion treatment. 

Among these 190 patients, 131 (68.9%) were seen at the time of their first-ever stroke. Ischemic strokes occurred most frequently, in 162 (85.2%) of the patients, while 20 (10.5%) had intraparenchymal hematoma, 3 (1.6%) transitory ischemic attack, 1 (0.5%) subarachnoid hemorrhage, 3 (1.6%) cerebral venous thrombosis and 1 (0.5%) ischemic and hemorrhagic stroke. The pathological subtype distribution of the ischemic strokes is shown in Table 2.

**Table 2.** Distribution of etiological subtypes of ischemic stroke according to the TOAST classification (N = 162).

Subtype	N	%
Large-artery atherosclerosis	36	22.2%
Small vessel disease	40	24.6%
Cardioembolism	15	9.2%
Other etiology	13	8.02%
Undetermined etiology	58	35.8 %
Incomplete	38	23.4%

The median NIHSS score was 2.5 (IQR, 1-5) and the mRS was 2 (IQR, 1-3). In the Bamford classification, we found that 84 cases (44.2%) were LACS, 77 (40.5%) were PACS, 11 (5.8%) were TACS, 15 (7.9%) were POCS and 3 (1.6%) were undetermined. 

With regard to neuroimaging, all the patients underwent parenchymal imaging, 138 (72.6%) brain computed tomography and 139 (73.2%) brain magnetic resonance imaging. Regarding vascular imaging, 132 (69.5%) underwent carotid and vertebral doppler ultrasound, 66 (34.7%) transcranial doppler ultrasound, 93 (48.9%) angioresonance of intracranial vessels, 52 (27.4%) angioresonance of cervical vessels, 20 (10.5%) angiography and 7 (3.7%) angiotomography. The most common abnormality was involvement of the middle cerebral artery region in 74 (54%), followed by multiple regions in 11 (15.3%). Patients also underwent other complementary examinations: 127 (66.8%) had an electrocardiogram, 85 (44.7%) Holter, 160 (84.2%) transthoracic echocardiogram and 15 (7.9%) transesophageal echocardiogram. 

One year after inclusion in the study, 12 patients (6.3%) had died and 14 (7.3%) had had another stroke. Table 3 describes the frequencies of non-pharmacological and pharmacological treatments. 

**Table 3.** Frequencies of non-pharmacological and pharmacological treatments.

	All patients (n = 190)	Ischemic stroke (n = 162)	Hemorrhagic stroke* (n = 21)	p-value
Speech therapy	27 (14.2%)	25 (15.4%)	2 (9.5%)	0.27
Physical therapy	70 (36.8%)	62 (38.2%)	7 (33.3%)	0.46
Occupational therapy	16 (8.4%)	16 (9.8%)	0 (0%)	-
Antihypertensive use	142 (74.7%)	118 (72.8%)	18 (85.7%)	0.02
Statin use	142 (74.7%)	125 (77.1%)	12 (57.1%)	-
Antiplatelet use	150 (78.9%)	138 (85.1%)	7 (33.3%)	-
ASA	112 (58.9%)	101 (62.3%)	7 (33.3%)	-
Clopidogrel	7 (3.6%)	7 (4.3%)	0 (0%)	-
ASA + clopidogrel	24 (12.6%)	23 (14.1%)	0 (0%)	-
Cilostazol	2 (1.05%)	2 (1.2%)	0 (0%)	-
Others	5 (2.6%)	5 (3.08%)	0 (0%)	-
Anticoagulant use	14 (15.5%)	12 (7.4%)	0 (0%)	-
Warfarin	10 (5.2%)	8 (4.9%)	0 (0%)	-
Dabigatran	2 (1.05%)	2 (1.2%)	0 (0%)	-
Rivaroxaban	2 (1.05%)	2 (1.2%)	0 (0%)	-

*For hemorrhagic strokes, only the cases of intraparenchymal hematoma and subarachnoid hemorrhage were considered; ASA: acetylsalicylic acid; ^#^(^2^ test: significance level < 0.05.

## DISCUSSION

This was, to the best of our knowledge, the first study to characterize stroke outpatients in Alagoas. Most of them were male and brown-skinned, and had a maximum income of three minimum monthly wages; 45% had low educational levels (maximum of four years). The most prevalent risk factors were sedentary lifestyle, hypertension and family history of stroke. 

An association between stroke and socioeconomic indicators had previously been described in the literature. Low socioeconomic status was correlated with a 67% increased risk of stroke^14^ and low educational levels were found to be an important predictor of functional dependence^15^ ^,^ ^16^. In the city of São Paulo, stroke mortality was found to differ among its districts according to their HDI, such that it was almost three times higher in the lowest HDI stratum of the city^17^. Alagoas has the lowest HDI of Brazil^18^ and the highest mortality rate among all northeastern state capitals^9^. Low socioeconomic status can be correlated with poor risk factor control^19^ ^,^ ^20^ and greater difficulty in accessing healthcare services, adequate acute treatment and post-acute care^21^. These factors directly affect patients’ prognoses and long-term survival^22^.

In Alagoas, the mortality rate has shown an increasing trend over recent decades^23^. Deaths caused by cerebrovascular diseases are concentrated in the eastern region of the state, probably caused by greater centralization of specialized healthcare services in the state capital, Maceió^24^. In 2018, only one stroke unit was available in the state, and this was located in the capital. In addition, also in the state capital, some private hospitals had stroke protocols. This scenario is insufficient for the whole population, and especially for people who do not live in the metropolitan region of Maceió, for whom no specialized stroke service is available. This situation can partially explain our reperfusion rate. Moreover, there is no defined flow of referrals from the stroke unit to specialized outpatient clinics after discharge, which therefore leads to delays in accessing investigative examination.

Other alarming findings were the low number of patients transported by ambulance to hospitals and the high number who sought a healthcare service with no imaging support. These results suggest that the population has poor knowledge about stroke. Pontes-Neto et al, 2008, showed that there was a lack of vital information in Brazilian population about stroke recognition and activation of the emergency medical services (EMS)^25^. In Alagoas, EMS are present throughout the state and there are 14 ambulances in the state capital. However, among our sample, the service was poorly activated. In Spain, being transported by an EMS vehicle was correlated with earlier arrival at the hospital and shorter door-to-imaging time, which is crucial for acute stroke management^26^. So far, few studies about prehospital care for stroke cases have been conducted in Brazil^27^. Such studies are necessary in order to understand people’s reasons for not requesting the EMS and the influence of this decision on the patient’s prognosis. 

Low-complexity emergency care units (ECUs) are an intermediate level healthcare service that form part of the national emergency care program in Brazil. They connect primary care, the EMS and tertiary-level care^28^. However, ECUs do not have imaging support and are not adequate for initial stroke treatment. In Fortaleza, Ceará, ECUs are the type of institution that is second most sought by patients with stroke symptoms. Age, educational level, sex and headache at onset, no speech deficits and prehospital transportation were previously found to be predictors of ECU utilization^29^. That study also indicated the importance of adequate training for ECU healthcare staff, to be able to rapidly recognize stroke symptoms and thus call out the EMS for referral of the case to hospital. Another matter is the importance of education for the population, so that stroke symptoms can be recognized and the EMS is then activated. 

In our sample, some patients were doing some kind of rehabilitation therapy, among which physiotherapy was undertaken most frequently (36.8%). In a previous study about functional outcomes among stroke patients in Alagoas, a functional dependence rate of 34.8% was found approximately two years after the ictus^30^. This was a high rate in comparison with other national studies^15^ ^,^ ^31^. Functional dependence may be related to greater stroke severity, the degree of access to adequate treatment during the acute phase and the availability of rehabilitation during the acute and post-acute phases^32^ ^-^ ^34^. Seventeen specialized rehabilitation services are currently available in Alagoas to attend to the 6% of this population that are declared to be disabled^35^ ^,^ ^36^. In addition to the insufficient number of rehabilitation services, the organization and quality of rehabilitation need to be considered.

No study has yet been conducted in Alagoas regarding the specialized stroke rehabilitation services in this state. However, certain factors contribute towards improving the quality of rehabilitation services, including systematic triage involving use of the International Classification of Functioning, Disability and Health (ICF); implementation of an individual therapeutic plan; frequent and systematic monitoring of functioning; and continuous education for the multidisciplinary team^37^. All of these aspects of the rehabilitation services need to be investigated in order to improve the rehabilitation process for stroke patients. 

Regarding etiology, we observed that the rate of undetermined strokes was 35.8%, and that for 23.4% this was because of incomplete investigation. A previous study conducted in Joinville, southern Brazil, found that the rate of undetermined etiology was 28.4%. In that study, all the patients underwent electrocardiography, extracranial and intracranial Doppler ultrasound, transthoracic echocardiography and at least one brain computed tomography. The high rate of undetermined strokes can be explained by the cryptogenic stroke included in this group^6^. 

Our stroke protocol for investigation of the event mechanism consists of laboratory tests, parenchymal imaging, study of intra and extracranial vessels and investigation of the cardiac routine (electrocardiogram and/or 24-hour Holter monitoring). However, in our state, the population has difficulty in accessing examinations within our public healthcare system and the low income of the study population does not allow these examinations to be performed within the private healthcare system. Similar results were found in Fortaleza, thus demonstrating the differences in access to healthcare services in Brazil^5^. These cases may be receiving inappropriate secondary prophylaxis and, consequently, there may be a higher chance of recurrence of cerebrovascular events.

Our study had some limitations. Our sample was restricted to patients attended in a specialized neurovascular clinic and our conclusion does not represent the reality of the entire state of Alagoas. The rates relating to examinations performed to investigate the stroke etiology, access to rehabilitation and recurrence may be worse overall because, in our scenario, patients are seen by a trained vascular neurologist and have easier access to examinations and treatments in the tertiary hospital. The majority of the patients included were chronic and we did not have access to all the data about the acute phase. As there is no referral flow of patients from acute-phase care services to outpatient clinics, we postulate that many more severely ill patients are unable to access outpatient care. Thus, further studies are necessary in order to understand the real situation of stroke treatment in Alagoas. 

In conclusion, our study was the first in Alagoas to characterize the clinical profile of a stroke sample, which is important, given the inequalities in Brazil. Our sample showed risk factors similar to those previously described in the literature, i.e. low educational level and low income. We also found that only a low number of patients were transported by ambulance to the hospital and that a high number of patients sought assistance at healthcare services with no imaging support, which suggested that the population has poor knowledge about stroke. The high number of cases of undetermined stroke etiology shows the difficulty in accessing healthcare services in our state and the urgent need for effective healthcare policies to improve the stroke care system.
